# A new coastal species of *Pseuderanthemum* (Acanthaceae) from Loyalty Islands (New Caledonia) and Vanuatu with notes on *P. carruthersii*

**DOI:** 10.3897/phytokeys.128.36325

**Published:** 2019-07-29

**Authors:** Gildas Gâteblé, Laurence Ramon, Jean-François Butaud

**Affiliations:** 1 Institut Agronomique néo-Calédonien (IAC), Equipe ARBOREAL, BP 711, 98810 Mont-Dore, New Caledonia Institut Agronomique néo-Calédonien Mont-Dore New Caledonia (Fr); 2 Botaniste associée à l’Université de Toliara, Madagascar Université de Toliara Toliara Madagascar; 3 Consultant in forestry and Polynesian botany, BP 52832, 98716 Pirae, Tahiti, French Polynesia Unaffiliated Pirae French Polynesia

**Keywords:** Acanthaceae, New Caledonia, new species, *
Pseuderanthemum
*, taxonomy, Vanuatu

## Abstract

When dealing with the taxonomy of Pacific coastal species within the region of New Caledonia and Vanuatu, one should examine all names published in Australasia and other Pacific islands. When the putative new species is also closely related to a highly praised ornamental species with many cultigens and with many old horticultural names, the task becomes more arduous. This is the case for the new species we describe as *Pseuderanthemum melanesicum* Gâteblé, Ramon & Butaud, which is closely related to the now pantropical cultivated species *P. carruthersii* (Seem.) Guillaumin s.l. Compared to *P. carruthersii*, *P. melanesicum* has carnose and shiny leaves, pedicels and sepals covered with glandular hairs, a short and enlarged corolla tube and can produce fertile capsules. The new species is a coastal taxon occurring naturally in the Melanesian archipelagos of New Caledonia and Vanuatu. This species seems uncommon in the Loyalty Islands but more common in the archipelago of Vanuatu and we propose it as Critically Endangered in New Caledonia, Vulnerable in Vanuatu and Least Concern when the IUCN evaluation is done globally.

## Introduction

The Pacific and Malesian taxa of Acanthaceae Juss. are in need of a broad taxonomic revision and especially those of *Pseuderanthemum* Radlk. (Barker 1986). *Pseuderanthemum* is traditionally defined as a pantropical genus with about 60 species both in the Old and New World tropics; the genus belongs to the tribe Justicieae of the subfamily Acanthoideae Link (Olmstead et al. 2016). The most recent and significant taxonomic treatments of the genus in the Pacific region are the ones by Heine (1976) for New Caledonia, Barker (1986) for Australia, and Smith (1991) for Fiji. There is presently also a lack of a good molecular phylogenetic framework for the genus in the region: few Pacific and Old World members of *Pseuderanthemum* have been included in the most recent phylogenetic and evolutionary studies (e.g. McDade et al. 2000, Daniel et al. 2008, Kiel et al. 2018). As currently circumscribed, *Pseuderanthemum* is not a monophyletic genus because the sampled New World, African and Asian species are placed in different clades (McDade, pers. comm.). Because the type species of the genus *Pseuderanthemum* is believed to be the New World *P. alatum* (Nees) Radlk. (Barker 1986), the Asian and Pacific taxa will need to be transferred to another genus once the molecular phylogenies will be better resolved based on a more comprehensive taxonomic sampling.

Some recent fieldwork and specimen collection conducted by the authors in the New Caledonian Loyalty islands (Lifou and Maré) and in Vanuatu led us to think that a taxon was missing in Heine’s (1976) full treatment of the genus in New Caledonia. After having looked at the many names already published in *Pseuderanthemum* and allied genera in the region to verify if a name was already available, we propose to describe it as a new taxon along with line drawings, color photos taken in the field, and an extinction risk assessment.

## Materials and methods

Most, if not all, published names of *Pseuderanthemum* and *Eranthemum* L. including some names in *Anthacanthus* Nees, *Chrestienia* Montrouz., *Graptophyllum* Nees, *Justicia* L., *Pachystachys* Nees, *Ruspolia* Lindau and *Siphoneranthemum* Kuntze said to be occurring in, or coming from, the central Indo-Pacific region were retrieved using International Plant Name Index (2012) and taxonomic publications and databases. Most of the protologues and type specimens (when scans were available) for these names were checked to know if they could be related to the new species. Careful examinations and measurements, in vivo, in the herbarium, and in alcohol, were conducted on morphological characters already used by Barker (1986) and Heine (1976) as taxonomically significant within the genus, to determine whether the species is new. Descriptions of color pertain to colors in vivo, unless otherwise noted. Descriptive terminology follows the glossary in Harris and Harris (2001). The species concept used here is based on morphological characters. Voucher specimens are deposited in K, LOY, MPU, NOU, NY, P and PVNH [abbreviations following Index Herbariorum (2019), except for LOY, which is the future herbarium code of the new Herbier de la province des Îles Loyauté].

## *Pseuderanthemum* s.l. in the Australasian and Pacific regions and domestication of *P. carruthersii*

From our bibliographic and type specimen image searches for about 100 *Pseuderanthemum* s.l. names in the region, it appears that the new species could only be confused with *P. carruthersii* (Seem.) Guillaumin. This latter species is, however, quite variable morphologically and widespread in cultivation throughout the tropics. In the tropics and in the Southwestern Pacific, especially Vanuatu and New Caledonia, *P. carruthersii* s.l. is a common garden ornamental showing a great morphological variability, especially in leaf size, shape and color. Like many other plants with variegated or colored leaves [e.g. *Abelmoschus manihot* (L.) Medik, *Acalypha wilkesiana* Müll.Arg., *Codiaeum variegatum* (L.) Rumph. ex A.Juss., *Cordyline fruticosa* (L.) A.Chev., *Dendrolobium umbellatum* (L.) Benth., *Graptophyllum pictum* (L.) Griff., *Hibiscus rosa-sinensis* L. s.l., *Pandanus tectorius* Parkinson ex Du Roi, *Plectranthus scutellarioides* (L.) R.Br., *Polyscias guilfoylei* (W.Bull) L.H.Bailey and *P. scutellaria* (Burm.f.) Fosberg], we believe that *Pseuderanthemum carruthersii* s.l. was selected and moved around during the migrations and peopling of the Melanesian islands well before European arrival in the region (Gâteblé 2015). It is then difficult to assess the true area of origin of these cultigens but most of them are coming from Southeast Asia or Papua New Guinea. Like most other such cultigens, *P. carruthersii* is a sterile (not fruiting) plant at least in Fiji (Smith 1991), New Caledonia and Vanuatu (authors pers. obs.) and is propagated through cuttings. Future molecular studies might inform about the wild relatives of the cultivated *P. carruthersii* s.l. as well as the peopling of the west Pacific as has been done for example in bananas (Perrier et al. 2011) and sweet potatoes (Roullier et al. 2013). To our knowledge, it is not known why such a large diversity of variegated and colored leaved plant species have been selected and spread through the Melanesian archipelagos but it seems to have played an important role in Melanesian culture and “gardens” (Gâteblé 2015).

## *Pseuderanthemum carruthersii* s.l., a taxonomically and nomenclaturally complex taxon

*Pseuderanthemum carruthersii* was originally described by Seemann (1866) as *Eranthemum carruthersii* from specimens collected by John MacGillivray on two islands (Aneityum and Erromango) in the south of the Vanuatu (former New Hebrides) archipelago, most probably in November and December 1853 (MacGillivray 1854). According to Smith (1991) there are three syntypes in BM (one without number from Erromango and two from Aneityum under *MacGillivray 30*) while Heine (1976) stated there are several specimens with at least the holotype in K and an isotype in BM. Both authors (Heine 1976, Smith 1991) were relying on a manuscript note from C.B. Clarke on the sheet from Erromango (BM001041151) to apply the name *E. carruthersii*. Smith (1991) chose this sheet (BM001041151) as the lectotype of the name *E. carruthersii* based on the note from Clarke describing this specimen with a short corolla tube of ca. 1 cm long. According to Clarke (in herb. and in Smith 1991), the two other syntypes *MacGillivray 30* from Aneityum in BM have a longer corolla tube (1.8–2.5 cm) and would represent a cultivated form of *E. carruthersii*. Also, Choopan et al. (2018) wrongly mentioned that Guillaumin (1948) designated the lectotype in BM.

Since *P. carruthersii* is a variable species and a highly regarded ornamental plant; it has been introduced multiple times from various sources and cultivated in European nurseries especially during the second half of the nineteen century. This has resulted in a plethora of names under *Eranthemum*, many of them being published in horticultural magazines several times with different authorships and, later on, combined or not under *Pseuderanthemum* and *Siphoneranthemum* by taxonomists. For most of the names, there is no herbarium specimen, the diagnosis is often short and/or incomplete and almost no lectotypification work has been done. It is not our intention to deal with the lectotypification and formal synonymization of all the names. Several authors have dealt, though incompletely, with the synonymization at the species or variety levels (see Fosberg 1955, Heine 1976, Fosberg and Sachet 1980, Smith 1991, Fosberg et al. 1993, Choopan et al. 2018) mainly depending on leaf shape and type of variegation.

Among all the names published so far, two are of particular interest for this work. The first one is the recently lectotypified *Pseuderanthemum maculatum* (G.Lodd.) I.M.Turner (Turner 2016) as, according to the designated lectotype (see illustration in Loddiges 1822), it is a reasonable match to *P. carruthersii* s.l. If this is the case, the name *P. maculatum* would have priority over *P. carruthersii*. The protologue does not give any indication about where the cultivated *P. maculatum* comes from but later Regel (1860) stated it comes from “*India orientalis*”. The second name is *Eranthemum marmoratum* W.Bull. According to the description of the vegetative parts of *E. marmoratum* in Bull’s horticultural catalogue (Bull 1874), this name might apply either to a cultigen of the new species or to a similar mottled-leaved *Graptophyllum pictum*. As there is no illustration or any herbarium specimen known to apply to the name *E. marmoratum*, it is best to consider this name as a doubtful one.

## Taxonomy of the new species

### Pseuderanthemum melanesicum

Taxon classificationPlantaeLamialesAcanthaceae

Gâteblé, Ramon & Butaud sp. nov.

9a3a8feb-c76d-5bc1-b9b3-c7d8b2981480

urn:lsid:ipni.org:names:60479324-2

Figs 1 , 2 , 3

#### Diagnosis.

*Pseuderanthemum melanesicum* Gâteblé, Ramon & Butaud is most similar to some cultigens of *P. carruthersii* but differs from them by its carnose leaf texture vs. chartaceous, by its pedicels and sepals with glandular hairs vs. eglandular hairs, by its glabrous petals and tube vs. hairy petals and tube and by its short and enlarged corolla tube vs. longer cylindrical corolla tube.

#### Type.

NEW CALEDONIA. Province des Iles Loyauté: Lifou, plant cultivated at the Agricultural Research Station Saint Louis at Mont-Dore, 4 December 2018, *G. Gâteblé 1072* (holotype P; isotypes NOU [NOU089981, NOU090339]).

#### Description.

Fruticulose shrubs up to 1.5 m tall, generally decumbent to somehow erect, somewhat carnose. *Branches* round (living material) to angulate (dry material) in cross section, beige to brown on older stems, pale green on young stems; prominent leaf and bundles scars; lenticels few, glabrous. *Leaves* simple, opposite-decussate, usually ovate to broadly elliptic, rarely obovate; blade (7–) 8–11 (–12) × (2.5–) 5–6 (–6.5) cm, vernicose, carnose, glabrous on both surfaces; apexes obtuse to rounded, sometimes slightly retuse or acuminate, bases cuneate to attenuate, margins entire; midveins slightly impressed adaxially, prominent abaxially toward the base, glabrous; secondaries of 4–6 opposite or alternate veins, more or less brochidodromous; tertiaries in a loose reticulum; petioles (0.5–) 1–1.5 (–2.5) cm long. *Inflorescences* terminal, a raceme or panicle, 1–12 cm long; rachises round to quadrangulate in cross section, mostly glabrous but with some glandular hairs in its most apical part; peduncles 1–5 cm long usually glabrescent but sometimes with some glandular hairs; bracts and bracteoles lanceolate 1–4 mm × 0.5–1.5 mm, glabrescent to piloglandulose. *Flowers* bilaterally symmetrical; pedicels 3–11 mm long, piloglandulose. Sepals 4 or 5, lanceolate, 3.5–4 × 1 mm, piloglandulose on the outer surface, glabrescent inside. Corollas ampliate to slightly ventricose, white with purple center, aestivation imbricate in bud; tubes ca. 1–1.3 cm long, glabrous, enlarged distally to 3–5 mm diameter before the throat, 4–5 lobed, consistently three in the lower half, and one or two in the upper half; lobes elliptic, 5–8 × 4–7 mm, the lower one being the larger, glabrous. Stamens 2, slightly exserted, inserted in the tube oriﬁce on to the upper lobe(s), filaments 4–4.5 mm long, glabrous, anthers ca. 1.8 × 0.8 mm; staminodes 2, ca. 1 mm long. Ovaries conical, 4.5 × 2 mm, glabrous; styles slightly exserted, ca. 15 mm long, glabrous; stigmas bilobed, lobes ca. 0.5 × 0.15 mm. *Fruits* stipitate dehiscent capsules, clavate, 1.5–3 × 0.6–0.8 cm, sometimes crowned with the remnant style; seed (4–?) per capsule, ovate, 3–4 mm × 2–3 mm.

#### Distribution and ecology.

**Figure 1.**
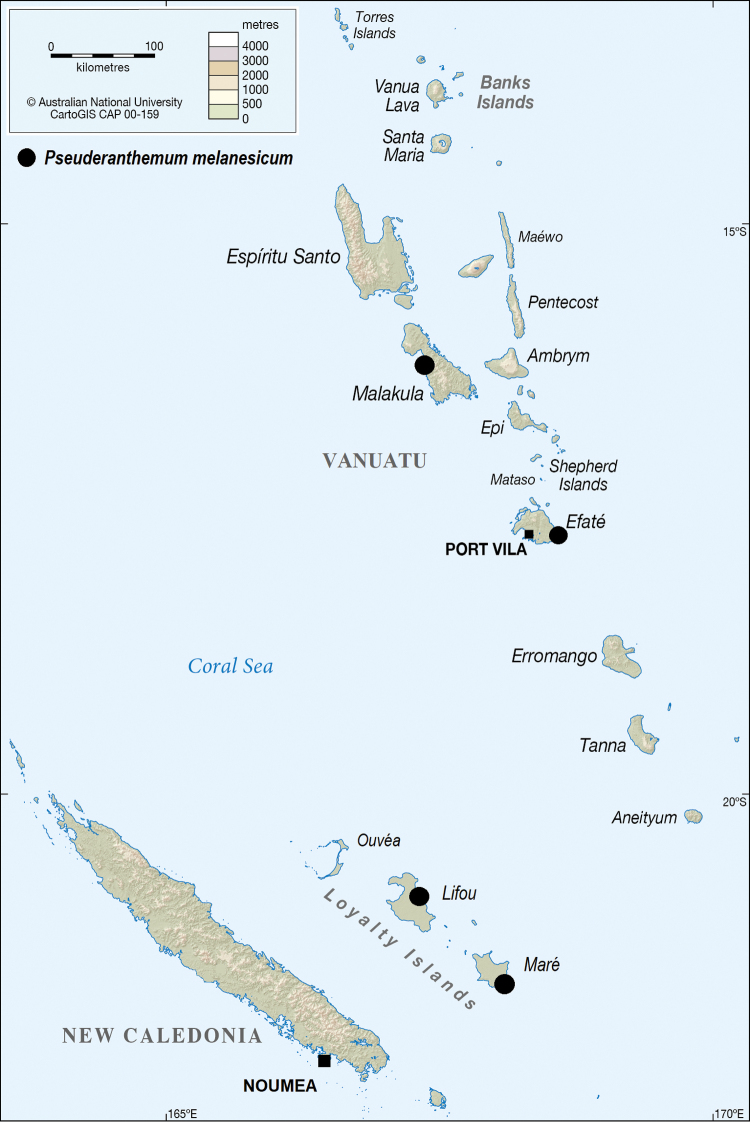
Distribution of *Pseuderanthemum melanesicum* Gâteblé, Ramon & Butaud, sp. nov. in some islands of New Caledonia and Vanuatu. Map done using CartoGIS Services, College of Asia and the Pacific, The Australian National University.

In New Caledonia and Vanuatu, *P. melanesicum* is found in coastal thickets on limestone substrate, either coastal reef, cliffs or back of the beaches, with species of *Bikkia* Reinw. ex Blume, *Dendrolobium* (Wight & Arn.) Benth., *Eugenia* P.Micheli ex L., *Heliotropium* Tourn. ex L., *Hibiscus* L., *Myoporum* Banks & Sol. ex G.Forst., *Nicotiana* L., *Pemphis* J.R.Forst. & G.Forst., *Sarcolobus* R.Br. and *Xylosma* G.Forst. at 2–60 m elevation in the Loyalty Islands. In New Caledonia, it is only known from Lifou and Maré in the Loyalty Islands and it is known from Efaté and Malakula in Vanuatu (Fig. 1). With such a distribution, the species should be more common than reflected by the available herbarium specimens. Recently (February 2019) the species was seen in relatively large populations on Erakor Island (Port Vila) and Port Resolution (Tanna). Like in other Acanthaceae, *P. melanesicum* seeds are dispersed through ballochory that could explain the many individuals found in some populations in Vanuatu. The seed seems also able to float on sea water for a few hours (observation made with only one seed).

**Figure 2.**
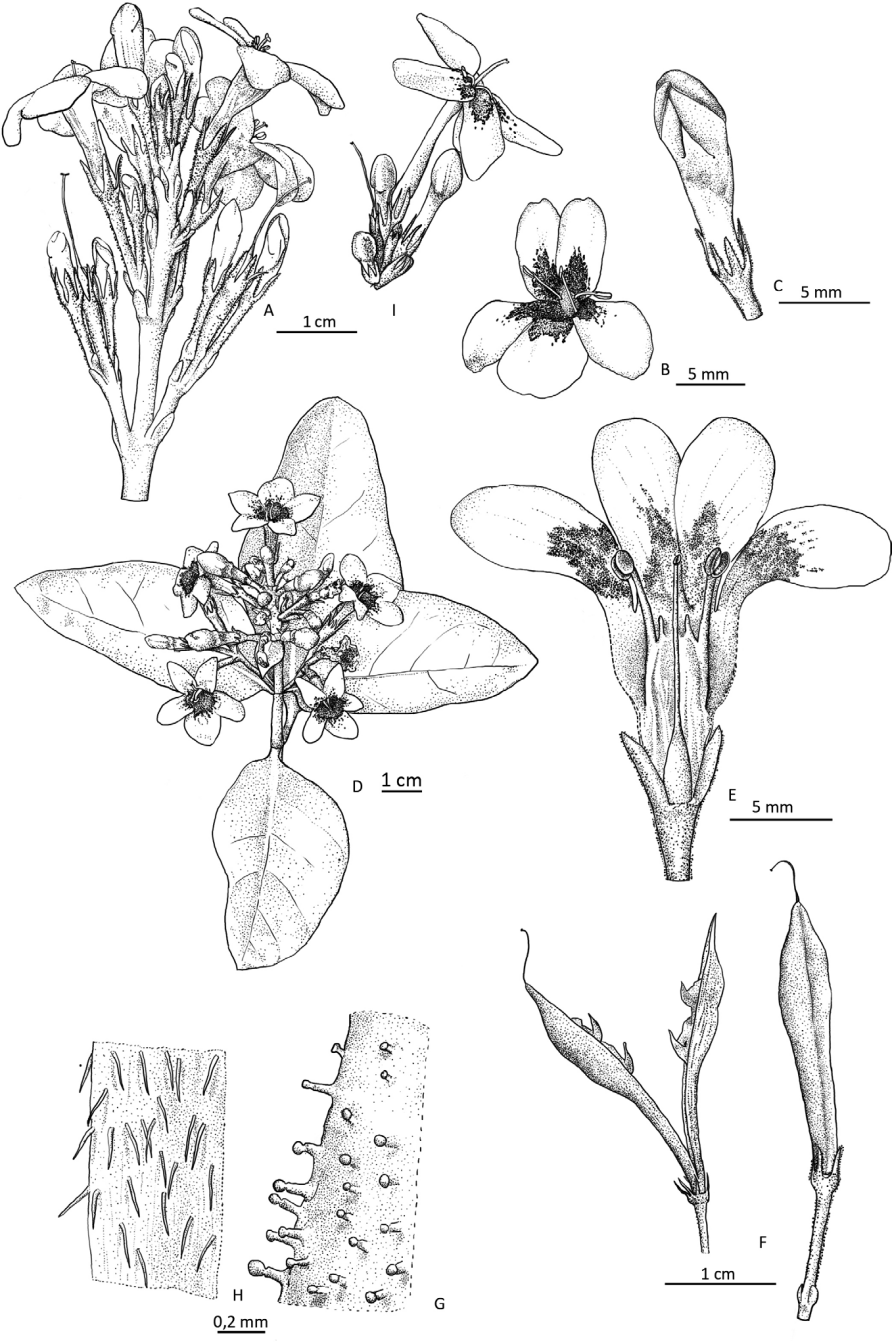
Drawings of *Pseuderanthemum melanesicum* Gâteblé, Ramon & Butaud, sp. nov. and *P. carruthersii***A–G***Pseuderanthemum melanesicum***H–I***Pseuderanthemum carruthersii***A, D** Structure of the inflorescence **B** Flower **C** Flower bud **E** Open corolla with the lower corolla lobe removed to show the arrangement of internal structures **F** Open mature and immature capsules **G** Glandular hairs on a flower bract **H** Eglandular hairs on the outer surface of the corolla tube **I** Part of inflorescence showing the long narrow tube of a flower. Voucher specimens: **A–D***Gâteblé 1072***E, G***Gâteblé 722***F***Ramon 220***H–I***Gâteblé 720*. Drawings by Laurence Ramon.

#### Etymology.

The new species is named after the Melanesian archipelagos of New Caledonia and Vanuatu.

#### Species recognition.

With its carnose and shiny leaves (especially seen on fresh material), its short and broaden corolla tube and its many glandular hairs on pedicels and sepals, *P. melanesicum* is easily separated from the cultivated, widespread and variable taxon, *P. carruthersii*. In addition, in both countries the new species has been collected in fruit while there is, to our knowledge, no fruiting specimen of *P. carruthersii* in the region. The well-known south-western Pacific botanical specialist Peter Shaw Green (1920–2009) also thought it was a putative new species as he wrote “*Pseuderanthemum* sp.? ined” on several herbarium sheets (e.g., *Hallé 6331* and *Gillison 3539*).

#### Notes.

**Figure 3.**
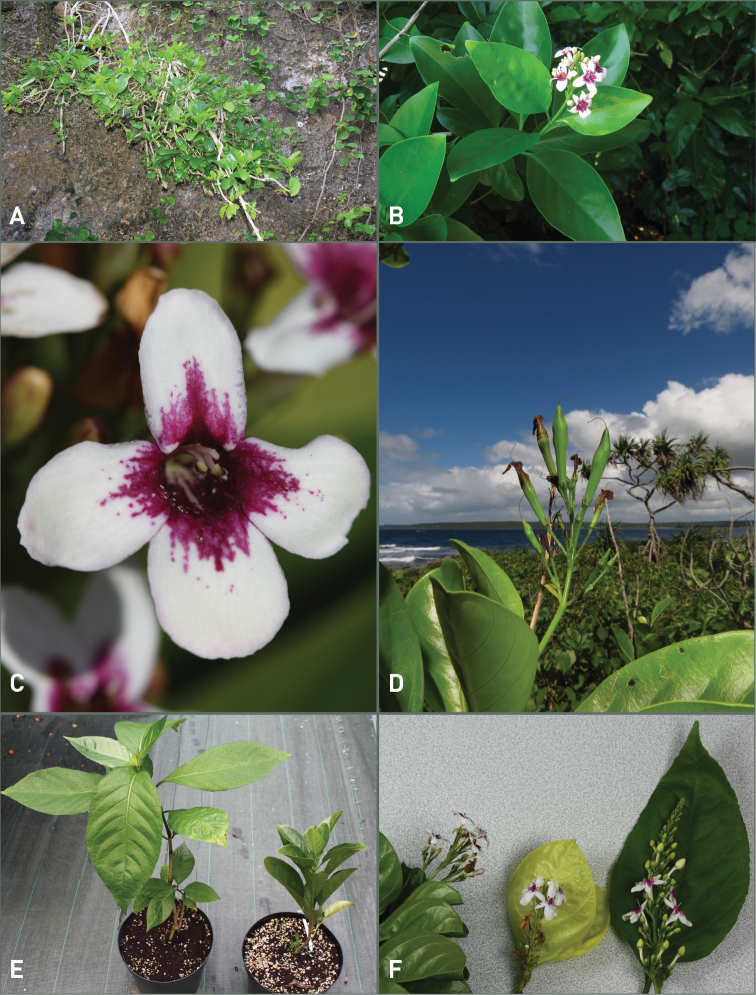
Field pictures of *Pseuderanthemum melanesicum* Gâteblé, Ramon & Butaud, sp. nov. and *P. carruthersii***A–D**. *Pseuderanthemum melanesicum***E–F***Pseuderanthemum melanesicum* and *P. carruthersii***A** Overview of a single shrub hanging from a coastal limestone cliff on Maré island **B** Flowering branch on Efaté island **C** Flower **D** Infructescence and ecology on Lifou island **E** Cultivated plants of *P. carruthersii* (left) and *P. melanesicum* (right) **F** Leaves and inflorescences of *P. melanesicum* (left) and two cultigens of *P. carruthersii* (center and right). Photographs by G. Gâteblé (**A, C, E–F**), L. Ramon (**B**) and J.-F. Butaud (**D**); Voucher specimens: **A***Gâteblé et al*. *1024*, **B***Ramon 220*, **C***Gâteblé 722*, **F***Gâteblé 722*, *721*, *720*.

Color figures of *P. melanesicum* have already been published twice under misapplied names of other species inhabiting Loyalty Islands and Vanuatu, once as P. repandum (G.Forst.) Guillaumin subsp. loyaltyensis (Guillaumin) Heine or «Waditcha» in Suprin (2008: 177), and the other one (fig. 119a) as *P. carruthersii* in Ramon and Sam (2015: 121). The vernacular name *Watija* in Maré can be related to P. repandum subsp. loyaltyensis but this name was not recorded recently (Lormée et al. 2011) for that species; *Watija* is clearly the local name of *Psychotria nummularioides* Baill. ex Guillaumin and has also been given to *Cleidion verticillatum* Baill., a shrub of the same size (Butaud, pers. obs.). Two local names are reported on *Gillison 3539* for Vanuatu as *Nuguvere* and *Malandi*. According to Gâteblé et al. (2018), *P. melanesicum* is only the third non-endemic species described from New Caledonia since the beginning of the 21^st^ Century. *Pseuderanthemum melanesicum* is easily propagated by cuttings and it thrives well in cultivation. Even in cultivation, the carnose and shiny leaves are maintained (Fig. 3) and it makes a nice native ornamental plant for gardens and landscaping in open or shaded areas.

The cultivated plant from which the type specimen was prepared was originally collected by J.-F. Butaud on Lifou, north of Wé to Luecila, (2 m elevation, 20°53'34.56"S, 167°16'18.56"E) on 19 April 2014.

#### Preliminary conservation status (IUCN 2017).

In New Caledonia, the species is very uncommon and was collected only recently from the east coasts of Lifou and Maré islands. Recently 15–20 shrubs were seen on Lifou and two on Maré but B. Suprin (pers. comm., 2015) states he has seen it in a few places on Maré and Lifou. On Maré, the species is threatened by feral goats that seem to graze young stems and that contribute to habitat degradation. On Lifou, in its only currently known location, the species is not clearly threatened; the only threats could come from agriculture as nearby areas are cultivated. The distribution of *P. melanesicum* in New Caledonia is similar to that of *Cyrtandra mareensis* Däniker but much rarer for the number of individuals. Its area of occupancy is 8 km^2^ while its extent of occurrence is less than 500 km^2^. It is considered severely fragmented as each subpopulation could go extinct with a very reduced probability of natural recolonization from the other subpopulation. A continuing decline has been observed and/or estimated for its quality of habitat and number of mature individuals. Based on the IUCN Red List Categories and Criteria (IUCN 2017) and using criteria B, *P. melanesicum* qualifies for Critically Endangered CR B2ab(iii,v) in New Caledonia. Using criteria D, with less than 50 individuals, *P. melanesicum* qualifies also for Critically Endangered (CR D). In Vanuatu, this species is commonly observed on Efaté island’s seashore and the major threats are forest and coastal clearing for housing on private properties (e.g. in Havannah and Undine bays and south from Eton village). The recently seen Erakor Island (Efaté) population is also facing disturbance due to the resort while the Port Resolution (Tanna) population is not facing major threat. No threat has been identified on Malakula and the species should also be present on other southern islands (Ambrym, Epi, Erromango and Aneityum) of the archipelago of Vanuatu towards the Loyalty Islands with no major associated threat. As a putative common species in Vanuatu with between five to ten locations and with threats only in the south of Efaté, the species could qualify as VU B2ab(ii,iii,v). Globally, for both New Caledonia and Vanuatu, *P. melanesicum* could be considered as Least Concern against IUCN criteria because of there being more than ten putative locations and both the extent of occurrence and area of occupancy are above the threshold for assessment of a threatened category using criterion B.

#### Additional specimens examined.

NEW CALEDONIA. Province des Îles Loyauté: Lifou, Luecila, 2 m, 20°53'34.56"S, 167°16'18.56"E, 15 April 2015, *J.-F. Butaud 3427* (NOU [NOU090338]); plant cultivated at the Agricultural Research Station Saint Louis at Mont-Dore, 13 May 2015, *G. Gâteblé 679* (NOU [NOU089984]); *ibid. loc*., 7 December 2015, *G. Gâteblé 722* (NOU [NOU089982, NOU089983]); *ibid. loc*. 29 December 2015, *G. Gâteblé 740* (NOU [NOU089985, NOU090341]); *ibid. loc*. 19 February 2019, *G. Gâteblé 1073* (NOU [NOU090340]). Maré: Sentier littoral entre Eni et Shabadran, 60 m, 21°39'25.57"S, 168°0'22.45"E, 2 April 2018, *G. Gâteblé*, *Drouin J*, *Jewine A. & Wamejongo W 1024* (MPU, NOU |NOU089986], P, K, LOY). VANUATU. Efaté, Eton, plage privée (avant le blue hole), 0 m, 17°45'0.55"S, 168°33'55.46"E, 5 August 2015, *L. Ramon 220* (NY [NY03487104], P [P02434405], PVNH). Malekula, Tisbel, 29 September 1971, *N. Hallé 6331* (NOU [NOU077567], P [P04385831]); *ibid. loc*., 28 September 1971, *A.N. Gillison 3539* (P [P04385834]).

## Supplementary Material

XML Treatment for Pseuderanthemum melanesicum
